# Prevalence of diabetes mellitus among non institutionalized elderly in Monastir City

**DOI:** 10.1186/1472-6823-12-15

**Published:** 2012-08-16

**Authors:** Sonia Hammami, Sounira Mehri, Said Hajem, Nadia Koubaa, Hala Souid, Mohamed Hammami

**Affiliations:** 1 Laboratory of Nutrition and Vascular Health, Faculty of Medicine, University of Monastir, Monastir, Tunisia; 2 Internal Medicine Department, University Hospital “F. Bourguiba”, Monastir, Tunisia; 3 National Institute of Public Health Tunis, Tunis, Tunisia; 4 Geriatric and Gerontology Unit, University of Ain Shams, Ain Shams, Egypt

**Keywords:** Diabetes, Elderly, Prevalence, Tunisia, Non institutionalized

## Abstract

**Background:**

Diabetes is a major public health problem worldwide. This problem is particularly relevant to the elderly. The prevalence of each condition increase with age. The present study aimed to determine the prevalence of Diabetes Mellitus (DM) among elderly; we also examined socio-economic factors and life style that are likely to be associated with DM.

**Methods:**

A cross-sectional study was conducted in 2008–2009, and used a multistage cluster sampling method to select a representative sample among non institutionalized elderly in Monastir City. A total of 598 elderly aged 65 to 95 years were included.

**Results:**

The prevalence of DM was 27.4% (29.2% in males’ vs 26.5% in females). Elderly with DM showed higher prevalence of hypertension, obesity and abdominal obesity. DM prevalence decreased with advancing ages in both men and women. Urban residents had a higher prevalence than did their rural counterparts. In multivariate analysis, DM was associated with abdominal obesity (OR [95% CI], 2.6 [1.1-6]; p <0.01), co-existing diseases (3.8 [2.4-6]; p <0.01), and hypertension (2.7 [1.6-4.5] ; p <0.01).

**Conclusion:**

The study highlights the DM problem in Tunisia. An ageing population together with social, economic and lifestyle changes have led to a dramatic increase in DM. These data emphasize the urgent need for a comprehensive integrated population-based intervention program to ameliorate the growing problem of DM.

## Background

Diabetes mellitus (DM) is a major public health problem which can cause serious complications, it’s amongst the five leading causes of death worldwide [1,2]. This problem is particularly relevant to the elderly. The prevalence of each condition increase with age. But the characteristic of the epidemiology of DM is the variability in the prevalence among different populations, especially in the elderly population. Previous surveys from Mediterranean older populations suggested that DM is present in epidemic proportions throughout the region as in Italy of 22.8% [3], in Crete of 27% [4], in Athens metropolitan region of 25.4% among males compared to 31% among females [5] and in Cyprus of 26% among males compared to 18% among females [6].

In Tunisia, despite an aging population, only a limited and fewer Tunisian studies have focused exclusively on the elderly. Tunisia is a country in transition. The elderly population aged more than 60 years has been rapidly increasing, from 5.5% in 1966 to 9.3% in 2004 of the country’s estimated population of 9910872 inhabitants. This percentage is expected to rise to almost 20% by the year 2040 [7]. Life expectancy at birth was 72.1 years in 2005, a figure that is envisaged to rise to 75.5 years in 2014 and nearly 77 years in 2029. However, the vast social and economic developments in Tunisia since 1956 have led to cultural changes including unhealthier eating habits, decreased physical activity and manifestation of a wide range of no communicable diseases [8]. DM is one of the most prevalent conditions (9% in the year 2005), and has become a health challenge in Tunisia [9]. According to World Health Organization’s, DM is one of the top continuing risk factors for cardiovascular disease in the world [2]. DM is one of the major contributors of metabolic syndrome due to its pathophysiological link to other cardiovascular risks, such as hypertension, dyslipidemia, obesity, unhealthier diet, sedentarity lifestyles and smoking habits [10].

The objective of this study was to investigate the prevalence of DM among elderly aged 65 years and older. We also examined socio-economic factors and life style that are likely to be associated with DM.

## Methods

### Study area and population

Monastir is a governorate located in the center ouest of Tunisia, 165 km from Tunis and includes 13 delegations. Monastir is a typical Mediterranean region with a total population of about 494900 inhabitants. The study was conducted in collaboration with World Health Organisation and the United Nations Population Fund. The data collection conducted between December 2008 and February 2009. The protocol for the study was approved by ethics committee of the University Hospital of Monastir and Ministry of health and all participants provided written informed consent. The present study is a part of the study undertaken in Monastir City to investigate social, health behaviours and health status of people aged more than 65 years and living in their home. The sampling methods were detailed elsewhere [11]. Briefly, Sample design was a representative cross sectional community-based study, using multistage, stratified, cluster sampling. The sampling scheme was designed by the National Institute of Statistics; stratification was made according to the 13 delegations and urban/rural environments. First we identified the districts, then we selected randomly a cluster of household with a probability of select proportional to the size of this population and all individuals aged more than 65 years were recruited into the sample. A total of 598 people aged more than 65 years living in their home in Monastir City were included in the analysis. Prior to screening, the participants were informed about the need of the study to maximise response rates. Twenty two persons selected refused to participate in the study given a non response rate of 3.7%. To facilitate data collection three medical doctors were recruited and trained on the study methodology. The questionnaire was developed, translated from French into Arab then pre-tested and validated in a pilot study by the National Institute of Public Health. The exclusion criteria were individuals aged less than 65 years and elderly present the day of the visit in the surveyed household found to be a visitor or do not usually leave there.

### Subject evaluation

Socio-demographic data (age, sex, marital and educational status). Information on habits (smoking, physical activity, activity of daily living), health status of elderly person were obtained via home-based personal interview. The structured questionnaire was followed by a physical examination to measure blood pressure, anthropometric variables and capillary glycemia (CG). DM was defined as those using medication for treatment of DM during the previous two week and who had previously informed by a medical doctor that they had DM [12]. Arterial hypertension was defined based on the JNC-7 cut-off point of 140 mmHg and above for systolic blood pressure and/or 90 mmHg and above for diastolic blood pressure [13], or using antihypertensive drug therapy in the previous 2 weeks. Body weight and height were directly measured using standard technique [14], Body Mass Index (BMI) was calculated as weight (in kilograms) divided by squared height (in meters squared). Participants were categorized according World Health Organization criteria, as normal (< 25 kg/m^2^), overweight (25–29.9 kg/ m^2^) and obese (≥ 30 kg/m^2^). Waist Circumference (WC) was measured with the tape midway between the lowermost rib margin and the iliac crest. Abdominal obesity was defined by a WC greater than 102 cm for men and greater than 88 cm for women [14]. CG was measured with a glucometer (Accu-Check) in a blood sample obtained from the fingertip. Participants were classified into two groups based on CG normal: CG <11.1 mmol/L or elevated with a CG concentration ≥11.1 mmol/L at screening [2,15]. CG greater or equal to 11.1 mmol/L was used for predicting undiagnosed diabetes in which diabetes was not diagnosed. Smokers were defined as those who were smoking at least one cigarette per day during the past year or not. Physical activity was defined as walking for at least 1 h per day or not. The diagnosis of dependency in Activity of Daily Living was made according to Katz scale and classified individuals as follows: a score of 6 points indicates full function, a score of 4 or 5 as moderate impairment and a score ≤ 3 indicates severe functional impairment [16]. Hospital admission over the year immediately preceding the survey was defined as a dichotomous variable: ≥one hospital admission in previous year or <1. Polypharmacy was defined as the use of three or more drugs recorded according to the Anatomical Therapeutic Chemical classification system [17]. We set the cut-off at three or more different drugs, because it is one of the most frequently used. Pills containing a fixed association of two active substances were counted as only one drug. Co-morbidities were defined as coexisting three or more chronic diseases.

### Statistical analysis

The data were extracted from “Epi data” and imported to SPSS 13 statistical software packages (SPSS Inc; http://www.spss.com). Results are expressed for continuous variables as mean ± standard deviation (M ± SD) and for qualitative variables as frequencies. Student t test or the Mann–Whitney U-test was used to estimate differences between mean values. Comparison of frequencies was performed by chi-squared test with the Yates correction when needed. Multiple logistic regression were used to examine the association between DM as a dependent variable and age, gender, hypertension, abdominal obesity, education, place of residence and co-morbidities as independent variables (a probability value of p <0.05 was considered statistically significant).

## Results

### Study sample characteristics

A total of 598 individuals (202 men and 396 women aged more than 65 years participated in the study. The mean age was 72.3 ± 7.4 years (range 65–95 years).

According to BMI 19.9% were normal, 31.1% were overweight and 49% were obese; according to WC cut-off points 73.2% had abdominal obesity.

### Prevalence of diabetes mellitus

DM prevalence was 27.4% (164/598). The prevalence was not different between men (29.2%, 59/202) and women (26.5%, 105/396). DM prevalence decreased with advancing ages in both men and women. Diabetic persons are younger than non diabetic persons (71.2 ± 6.9 vs 72.6 ± 7.2 years, p = 0.04). Urban residents had a higher prevalence than did their rural counterparts. The prevalence of DM rose with in increase in educational level in both men and women, but the difference was not significant. The prevalence was not different between married and single (29.8% vs 22.2% for men) and (27.4% vs 25.7% for women), Table 1.

**Table 1:** Prevalence of diabetes mellitus among women and men according to socio-demographic characteristics

	**Male**		**Female**	
	**n**	**%**	**n**	**%**
Age (years)				
<70	19/57	33.3	54/170	31.7
70-74	16/53	30.1	22/89	24.7
75-79	14/52	27.2	14/70	20.0
≥80	10/40	25.0	15/67	22.3
p-value	0.8		0.1	
Administrative area				
- Urban	54/169	32.5	97/347	27.9
- Rural	5/33	15.1	8/49	16.3
p-value	<0.01		0.1	
Education				
- <Primary	42/153	27.4	99/379	26.1
-Primary or higher	17/49	34.6	6/17	35.2
p-value	0.1		0.6	
Marital status				
- Married(e)	55/184	29.8	48/175	27.4
- Single	4/18	22.2	57/221	25.7
p-value	0.6		0.7	

Associations between DM and risk factors are presented in Table 2. Men and women with higher BMI and abdominal obesity had a higher prevalence of DM. The BMI and WC were significantly higher in diabetic participants than in non diabetic subjects (32.4 ± 7 vs 29.4 ± 6 kg/m^2^, p <0.001; 107.7 ± 12 vs 101 ± 12 cm, p <0.001, respectively). Table 2 showed a strong association between hypertension and DM. Men and women with hypertension had a higher prevalence of DM. The physical activity, dependency and smoking status (results not showing) of the interviewees did not present any association with DM. Glycemic control was higher in diabetic participants, CG ≥11.1 mmol/L was present in 52% of the individuals with DM and 5.5% of non diabetic participants, p <0.0001. Of those who are non diabetic, 5.5% (24/434) had a CG ≥11.1 mmol/l. In both sexes, the prevalence of DM increased progressively with the number of drugs used and coexisting illnesses. However, the number of hospital admission in preceding year is positively but not significantly associated with DM (Table 2).

**Table 2:** Prevalence of diabetes mellitus among women and men according to cardiac risk factors and lifestyle in participants

	**Male**		**Female**	
	**n**	**%**	**n**	**%**
**Overweight (kg/m**^**2**^**) (n = 531)**				
BMI:<25	6/58	10.3	7/48	14.5
BMI : 25–29.9	17/70	10.0	23/95	24.2
BMI:≥30	25/52	48.0	65/208	31.2
p-value	<0.001		<0.05	
**Abdominal obesity**				
**(n = 531)**				
No	26/108	24.0	5/34	14.7
Yes	25/72	34.7	90/317	28.3
p-value	<0.01		<0.05	
**Capillary Glycemia (mmol/L)**				
< 11	22/159	13.8	44/317	13.8
≥ 11.1	37/43	86.0	61/79	81.3
p-value	<0.001		<0.001	
**Hypertension**				
Yes	36/91	39.5	80/220	36.3
No	22/111		26/176	
p-value	<0.01	19.8	<0.001	14.7
**Physical activity**				
Yes	4/16	25.0	2/7	28.5
No	55/186	29.5	103/389	26.4
p-value	0.9		0.7	
**Dependency**				
Severe functional impairment	9/20	45.0	12/37	32.4
Moderate impairment	14/40	35.0	31/110	28.1
Full function	36/142	25.3	62/249	24.8
p-value	0.3		0.5	
**N° of hospital admission in preceding year**				
≥ 1	15/41	36.5	22/68	32.3
<1	43/161	26.7	83/328	25.3
p-value	NS		NS	
**Comorbidities**				
No	28/147	19.1	44/278	15.8
Yes	30/55	54.5	62/118	52.5
p-value	<0.001		<0.001	
**N° of drugs**				
< 3 drugs	14/95	14.7	13/142	9.1
≥3 drugs	45/107	42.0	92/254	36.2
p-value	<0.001		< 0.001	

N°: number.

In multivariate analysis, DM was associated with abdominal obesity (OR [95% CI], 2.6[1.1-6]; p <0.01), co-morbidities (3.8 [2.4-6]; p <0.01), and hypertension (2.7 [1.6-4.5]; p <0.01) (Table 3).

**Table 3:** Logistic regression models of predictors of awareness of DM among adults aged 65 and older in Monastir City (n = 531)

**Variable**	**Odds ratio**
**Abdominal obesity**	
No	1.00
Yes	2.61 ; *1.1- 6*^a^
**Comorbidities**	
No	1.00
Yes	3.80 ; *2.4- 6*
**Hypertension**	
No	1.00
Yes	2.7 ; *1.6-4.5*

Variables included in the model at the first step but which were not statistically significant included , gender, age, education, place of residence (urban vs rural).

^a^ Figures in italics are the 95% confidence intervals.

## Discussion

The data obtained from this community based study report an overall prevalence of DM of 27.4% (164/598) in Monastir City. Clearly, a substantially higher prevalence than that has been reported by previous study for Tunisia [18,19]. Compared to other surveys that used WHO diagnostic criteria, prevalence of DM in elderly living in their home in Monastir City is higher than in Thailand [20], Nepal [21], Greece [22], Spain [23], French [24], and Canadian [25], lower than in Egypt [26], in Saudi Arabia [27], United Arab Emirates, Bahrain and Oman [28], India and Singapore [29,30], and similar to that in US [31], and Mayotte [32]. The observed increase in DM prevalence obtained from our study is likely to be explained by several factors. First, our subjects are selected in older age group (more than 65 years), as it is well known that diabetes prevalence increases with age [2]. Second, another possible reason is the fact that risk factors for developing DM are also increasing in Tunisia, particularly, obesity and sedentary life with lack of physical activity [8]. Third, the diagnosis of DM obtained from our study, was based on the new criteria endorsed by the ADA and adopted by WHO in 1998 [33]. The possible other causes to explain higher prevalence of DM in the centre region is probably urbanization (more than 80% individuals live in urban areas) with less physical activity and higher rates of obesity. The data obtained from our study indicate a rural/urban differences in prevalence with a higher prevalence of DM among Tunisian living in urban areas of 29.2% compared to rural Tunisian’s of 15.9% (p <0.001). Rural populations with more traditional lifestyles exhibit lower rates of DM risk factors and DM, whereas urban populations, and particularly those of a higher socioeconomic status, have a higher rate of both risk factors and DM. It is clear that various environmental and lifestyle factors can increase the likelihood that a genetically susceptible individual will develop diabetes in old age.

Furthermore, apparently, the reported figures from this study is alarming, demonstrating an epidemic of DM striking the Tunisian elderly population. Our study showed a higher prevalence of DM among males of 29.6% compared to 26.5% in females. DM prevalence increased with age, peaking among those less than 70 years of age and then decreasing. The age-and gender- specific prevalence of DM are relatively similar to those of several cohorts [26,27,34]. However the present study found that the DM prevalence declined after age 70 years. It is possible that the earlier onset of DM with a high prevalence and a high incidence of cardiovascular disease and myocardial infarction resulted in a greater premature mortality in diabetic individuals, and that this contributed to the decline in the prevalence of diabetes after 70 years of age in Tunisian subjects [11,25]. It is no clear whether insulin clearance or genetic background played a role. The proportions (5.5%) predicting undiagnosed DM, were relatively lower than those of some developing countries [26,27]. WHO currently recommends that diabetes be diagnosed by measuring venous fasting blood glucose [33], in this study, in view of its practical application, agility, rapidity and safety we used CG for identifying undiagnosed DM. The CG test is considered to be a convenient screening method in low resource communities. However the discriminatory capacity would like be improved by a second confirmatory venous blood glucose test [15,35].

The DM prevalence increased in those with higher educational level in both women and men, but the difference was not significant, this is consistent with the findings from other developing countries [20], but is in contrast to the developed countries, where the prevalence was higher in those with a lower educational level [36]. Confirming the literature data, the study revealed that several cardiovascular risk factors such as hypertension, obesity and abdominal obesity are more common in diabetic than non diabetic subjects. Abdominal obesity and hypertension were the main predictors for DM, even after adjustment on potential confounding factors. The present study showed that 48% of male Tunisian with obesity (BMI ≥30 kg/m^2^), studied in this survey are diabetic demonstrating an association between higher rates of DM with obesity. This association was also evident among female subjects. The positive relationship between obesity and DM was well recognized [37-39]. It is well documented that obesity is a strong risk factor for the development of DM as shown by several studies [40,41]. There is also an age related increase in obesity, particularly central obesity, and a reduction in physical activity, both of which can be associated with abnormal glucose metabolism. According to Diabetes Prevention Program, for every kilogram of weight loss, there was a 16% reduction in risk, adjusted for changes in diet and activity [42]. In fact, the Diabetes Prevention Program found that modifications of diet and activity were more effective in older people than in younger patients in preventing the development of DM. It is believed that obesity contributes to the development of DM by elevating levels of non esterified free fatty acids, hormones, adipocytokines, and other substances that increase insulin resistance. Obesity-related elevation in proinflammatory molecules, including tumor necrosis factor-α and interleukin-6 are also believed to contribute to the development of both DM and metabolic syndrome [43,44]. The prevalence of overweight and obesity among patients with DM are both extremely high. Obesity or abdominal obesity is frequently associated with other cardiometabolic factors such as hypertension, lipid disorders. These factors may be clustered as metabolic syndrome, which is well known to seriously increase the cardiovascular risk [45,46]. In the present study, those with diagnosed DM tended to have a higher prevalence of high blood pressure in both men and women. Individuals with DM had more concomitant cardiovascular risk. The rapid transition and changes of lifestyles involving dietary intake (high fat caloric dense food, low in complex carbohydrates, refined sugar and salt) and decreasing physical activity have take a place in the past 20 years in Tunisia. The urbanization had negative consequences on lifestyle aspects of Tunisians [8]. This suggests that there is a need to improve the awareness of health and use of regular activity and Mediterranean diet to prevent or reduce the cardiovascular risk. For those reasons, the promotion of healthier eating habits may protect elderly and contribute to improved health and quality of life. The Mediterranean diet is a healthy dietary pattern that has already shown its cardioprotective effects [47]. The Mediterranean diet is characterized by high consumption of monounsaturated fatty acids (Olive oil), and encourages daily consumption of fruits, vegetables, whole grain cereals with low animal-fat consumption. Recently, a meta-analysis, performed in 50 studies, with an overall incorporated population of approximately one-half million subjects, revealed the beneficial effect of the Mediterranean diet. The researchers showed that adherence to the Mediterranean diet have a positive effect on human health through the beneficial effect on abdominal obesity, lipids levels, glucose metabolism and blood pressure. Results indicate that adherence to Mediterranean diet may attenuate the prevalence of metabolic syndrome and, consequently the increasing burden of DM and cardiovascular diseases [48]. Pathophysiological mechanisms include antioxidant and anti-inflammatory effects of the functional foods included in the Mediterranean diet [48].

Physical activity is well recognized as cardiometabolic protect factor [6]. However, physical activity was not associated with DM in our study. This surprising finding may be explained by the small number of elderly who practice regular physical activity (less than 4%). Increasing numbers of diseases and drugs are associated with high prevalence of DM. The number of hospital admission in preceding year was also positively associated with DM. Elderly diabetic patients often require multiple medications to adequately and appropriately treat their diabetes and associated comorbidities. It is also, well documented that co-existing illnesses and take multiple drugs can adversely impact glucose metabolism [49].

It is important to acknowledge that estimation of the prevalence of DM in elderly population and formulation of effective treatments strategies is only the first step. It is clear that the magnitude of the problem of DM among the elderly poses a major economic challenge to health care systems. We believe that higher public health organization, health planners, clinicians and public health practitioners in developing countries must formulate a program targeting high risk group to implement a preventive strategy in modifying lifestyle by increasing physical activity, reducing obesity, adopting healthier eating habits and raising diabetic’s awareness of their disease to reduce morbidity and mortality [50].

### Limitations

This study has some limitations. First, the use of self-report data to estimate the prevalence of diabetes is problematic, as it potentially undercounts those in whom diabetes has not been diagnosed and over-counts those wrongly said to have diabetes. Our estimates would therefore be conservative. Second, we did not perform plasma glucose measures tests, which are more sensitive than random capillary glycemia and thus would have resulted in a higher estimate of previously unrecognized diabetes. In addition, our epidemiological estimates of unawareness of diabetes states were based on a single measurement of CG, which may have led to overestimation and should be interpreted with caution. Another limitation of our study is related to the cut-off values used for the BMI, WC, which were established in studies mostly performed in Caucasian populations and, thus, may or may not be valid in Tunisian elderly population. The strengths of this study include its population-based sampling frame and the availability of clinical measurements, To our knowledge, this is the first study in Tunisian population to report the prevalence of DM and risk factors among adults 65 years and older. Such information may be helpful to health care decision makers.

## Conclusions

DM is very common in elderly Tunisian subjects and is frequently associated with other cardiovascular risk factors, mainly obesity and hypertension. An ageing population together with social, economic and lifestyle changes have led to a dramatic increase in DM. These data emphasize the needs to inform the general public and health planner. There is an urgent need for a comprehensive integrated population-based intervention program to ameliorate the growing problem of DM. The above information suggests that lifestyle modifications might be of value in the DM prevention in older people.

## Abbreviations

DM, Diabetes Mellitus; CG, Capillary Glycemia; WC, Waist Circumference; BMI, Body Mass Index.

## Competing interests

The authors have no conflict of interest to declare.

## Authors’ contributions

SH contributed to the conception and design of this study, general supervision of the research group, interpretation of data, and drafting the manuscript. SM contributed to analysis and revising the article critically. SHa contributed to the conception and design of this study, analysis and interpretation of data. NK participated in the recruitment of study subjects and collection epidemiological data HS revised the manuscript critically for important intellectual content. MH revising the article critically for important intellectual content. All authors read and approved the final manuscript.

## Pre-publication history

The pre-publication history for this paper can be accessed here:

http://www.biomedcentral.com/1472-6823/12/15/prepub
